# Emergence and potential for spread of Chikungunya virus in Brazil

**DOI:** 10.1186/s12916-015-0348-x

**Published:** 2015-04-30

**Authors:** Marcio Roberto Teixeira Nunes, Nuno Rodrigues Faria, Janaina Mota de Vasconcelos, Nick Golding, Moritz UG Kraemer, Layanna Freitas de Oliveira, Raimunda do Socorro da Silva Azevedo, Daisy Elaine Andrade da Silva, Eliana Vieira Pinto da Silva, Sandro Patroca da Silva, Valéria Lima Carvalho, Giovanini Evelim Coelho, Ana Cecília Ribeiro Cruz, Sueli Guerreiro Rodrigues, Joao Lídio da Silva Gonçalves Vianez, Bruno Tardelli Diniz Nunes, Jedson Ferreira Cardoso, Robert B Tesh, Simon I Hay, Oliver G Pybus, Pedro Fernando da Costa Vasconcelos

**Affiliations:** Center for Technological Innovation, Evandro Chagas Institute, Ministry of Health, Ananindeua, PA 67030-000 Brazil; Department of Zoology, University of Oxford, South Parks Road, Oxford, OX1 3PS UK; Spatial Ecology and Epidemiology Group, Department of Zoology, University of Oxford, Oxford, OX1 3PS UK; Department of Arbovirology and Hemorrhagic Fevers, Evandro Chagas Institute, Ministry of Health, Ananindeua, PA 67030-000 Brazil; National Dengue Control Program, Brazilian Ministry of Health, Brasilia, DF 70058-900 Brazil; Department of Pathology, University of Texas Medical Branch, Galveston, Texas TX 77555-0609 USA; Fogarty International Center, National Institutes of Health, Bethesda, MD 20892 USA; Department of Pathology, Para State University, Belem, PA 66087-670 Brazil

**Keywords:** Chikungunya virus, National surveillance, Public health, Spatial prediction, Statistical methods

## Abstract

**Background:**

In December 2013, an outbreak of Chikungunya virus (CHIKV) caused by the Asian genotype was notified in the Caribbean. The outbreak has since spread to 38 regions in the Americas. By September 2014, the first autochthonous CHIKV infections were confirmed in Oiapoque, North Brazil, and in Feira de Santana, Northeast Brazil.

**Methods:**

We compiled epidemiological and clinical data on suspected CHIKV cases in Brazil and polymerase-chain-reaction-based diagnostic was conducted on 68 serum samples from patients with symptom onset between April and September 2014. Two imported and four autochthonous cases were selected for virus propagation, RNA isolation, full-length genome sequencing, and phylogenetic analysis. We then followed CDC/PAHO guidelines to estimate the risk of establishment of CHIKV in Brazilian municipalities.

**Results:**

We detected 41 CHIKV importations and 27 autochthonous cases in Brazil. Epidemiological and phylogenetic analyses indicated local transmission of the Asian CHIKV genotype in Oiapoque. Unexpectedly, we also discovered that the ECSA genotype is circulating in Feira de Santana. The presumed index case of the ECSA genotype was an individual who had recently returned from Angola and developed symptoms in Feira de Santana. We estimate that, if CHIKV becomes established in Brazil, transmission could occur in 94% of municipalities in the country and provide maps of the risk of importation of each strain of CHIKV in Brazil.

**Conclusions:**

The etiological strains associated with the early-phase CHIKV outbreaks in Brazil belong to the Asian and ECSA genotypes. Continued surveillance and vector mitigation strategies are needed to reduce the future public health impact of CHIKV in the Americas.

**Electronic supplementary material:**

The online version of this article (doi:10.1186/s12916-015-0348-x) contains supplementary material, which is available to authorized users.

## Background

The Chikungunya virus (CHIKV) is a re-emerging mosquito-borne alphavirus posing a significant public health problem in tropical and subtropical regions [1]. CHIKV infection is usually characterized by an acute onset of fever, rash, and arthralgias, and is often accompanied by headache, joint swelling, and conjunctivitis. Unlike dengue virus (DENV), CHIKV infection is associated with recurrent polyarthralgias and high rates of symptomatic infections. CHIKV is typically transmitted between humans by the anthropophilic vectors *Aedes aegypti* and *Ae. albopictus* [2].

Four CHIKV genotypes have been identified since its discovery in 1952 [3]. The East-Central-South-African (ECSA) and West African genotypes are endemic and cause epidemics in sub-Saharan Africa, whereas the Asian genotype circulates in *Ae. aegypti*-human urban transmission cycles in Southeast Asia. The Indian Ocean lineage (IOL) caused explosive epidemics in Indian Ocean islands and Asia between 2005 and 2011. Several IOL strains harbour mutations that augment infectivity and transmissibility in *Ae. albopictus* [4]. Imported cases caused limited outbreaks in temperate regions where only *Ae. albopictus* is present [5]. On December 5, 2013, Asian genotype CHIKV infections were reported in the Caribbean island of Saint Martin [6]. For the first time in recorded history, CHIKV had established a mosquito-human cycle in the Americas. As of October 31, 2014, >1,222,000 suspected CHIKV cases have been reported in the Americas and autochthonous infections have been confirmed in 50 territories in the region [7].

The first autochthonous cases of CHIKV in Brazil were confirmed in Oiapoque, Amapa State, on September 13, 2014. Seven days later, autochthonous cases were also confirmed in Feira de Santana, Bahia state. By October 18, 2014, 682 confirmed autochthonous cases had been notified to the Brazilian Ministry of Health [8]. Yet, the source of the ongoing outbreaks in these municipalities and the potential for virus establishment in the country remain unknown. We performed serological and virological analysis on 68 samples collected from June to September 2014 and full-length viral genome sequencing on six isolates. CHIKV and DENV have similar transmission cycles [9], and DENV is hyperendemic in Brazil [10]. Herein, we describe the epidemiological and genetic characteristics of CHIKV emergence in Brazil and provide a prediction of the risk of CHIKV importation and establishment in each Brazilian municipality for the coming year, using data on human mobility, vector distribution, and retrospective DENV incidence.

## Methods

### Patients and diagnostic assays

Serum was sampled from 68 patients exhibiting symptoms consistent with CHIKV infection and detected through a passive surveillance national system. Demographic data included patient age, sex, municipality, and federal state where the case was reported, date of onset of symptoms, date of sample collection, history of travel and residence, plus CHIKV and DENV serology. Data were provided by the Evandro Chagas Institute and the National Program for Dengue Control, Department of Surveillance in Health, Brazil. Samples were sent to the Evandro Chagas Institute for confirmation of CHIKV infection using IgM ELISA, real-time PCR, and C6/36 cell culture. Samples were obtained from persons visiting local clinics by Ministry of Health personnel as part of dengue surveillance activities. In these situations, patient consent is oral and not recorded. The study was authorized by the Coordination of the National Program for Dengue Control run by Brazil’s Ministry of Health.

### Virus propagation and RNA isolation

CHIKV isolates were recovered from sera of six acutely infected patients (P25, P34, P36, P37, P38, P39; Additional file 1: Text S1 and Table S1) in low-passage monolayer cultures of *Ae. albopictus* C6/36 cells, harvested 6 to 10 days post infection or after evident cytopathic effect. Culture suspensions were centrifuged and treated with 50% polyethylene glycol 8000 and 23% NaCl for virus precipitation. After centrifugation, virus pellets were eluted in RNase-free water and used for RNA isolation. Samples were subjected to a TRIzol separation step and the aqueous phase was further processed using Qiamp Viral RNA Minikit (Qiagen) according to the manufacturer’s instructions.

### Viral genome sequencing

Viral genomes were recovered using the Ion Torrent PGM [11]. Briefly, the cDNA synthesis reaction was performed using an in-house protocol with random primers. Samples were fragmented by enzymatic digestion and libraries built by the automated AB Library Builder System (Applied Biosystems). To normalize the number of molecules required for emulsion PCR, a quantitation step was performed using the automated Ion OneTouch 2 platform. The emulsion PCR reaction result comprising barcoded-pooled samples was loaded on a 318v2 chip and the sequencing reaction was performed on the PGM device. Raw reads were assembled using Mira v4.0 [12] (Additional file 1: Text S1). The six CHIKV genomes generated here have GenBank accession numbers: KP164567–KP164572.

### Phylogenetic analysis

To investigate the origins of CHIKV in Brazil, 155 publicly available genomes (>10,000 nt, by June 2014) with known dates and locations of sampling were collected from GenBank [13] and added to the new Brazilian genomes. Sequences were aligned using MAFFT [14]. Viral phylogenies based on full-length nucleotide sequences were estimated using maximum likelihood implemented in PhyML [15] and using BEAST [16], a program for Bayesian analysis of molecular sequences using Markov Chain Monte Carlo (MCMC). The GTR + 4Γ + I substitution model was used for phylogenetic reconstruction, as suggested by jModelTest [17]. After excluding 85 IOL strains, a total of 76 CHIKV genomes representing all four viral genotypes were used. Specifically, the final full-length dataset of 76 full-length genomes included 11 West African, 12 ECSA, 17 IOL, and 30 Asian epidemic strains, plus 6 new Brazilian strains (Accession Numbers in Additional file 1: Text S1). A maximum likelihood analysis was also performed using an E1 envelope alignment including all 554 available sequences for which the date and country of collection were available (Additional file 1: Figure S2). For the full-length dataset, a relaxed lognormal molecular clock model [18] and the non-parametric skygrid coalescent model [19] were employed. Using a strict molecular clock model and the skyline coalescent model [20] produced similar results. Six runs using the full-length dataset were computed for 50 million MCMC steps and convergence inspected using Tracer v.1.6 [16]. A maximum clade credibility phylogeny was obtained from the combined posterior distributions of the full-length dataset runs (excluding 10% burn-in) using TreeAnnotator [16]. Similar analyses were also computed separately for each genotype. For each estimated parameter, we report the mean and the corresponding uncertainty as 95% Bayesian credible intervals (BCI).

### Predicting importation and establishment risk

Given the poor dispersal ability of *Ae. aegypti* and *Ae. albopictus* [21], introduction of CHIKV in Brazil will largely be driven by human movements. We therefore predicted the risk of CHIKV importation into each Brazilian municipality using a radiation parametric model of human mobility with selection, which has shown strong predictive power at sub-national scales [22]. Empirical data concerning short-term human movements (as opposed to long-term relocations) were unavailable for Brazil or surrounding countries, hence model parameters were estimated using a previously published directed commuting network for France, obtained from 5,695,974 anonymized mobile phone records comprising directional movements among 329 districts [23]. Population sizes in each of these districts and in Brazilian municipalities were extracted from the Gridded Population of the World, v3 dataset [24] using administrative boundary data from the GADM database [25]. Parameters were estimated by maximum likelihood using a standard numerical optimisation routine [26] in R software v3.1.1 [27] and applied to predict population-level movements between 5,494 municipalities in Brazil (R code available on request). For each municipality, we included the presence of *Ae. aegypti* and *Ae. albopictus* recorded from annual larval surveys conducted between 2007 and 2014 [28], or of reported DENV transmission between 2001 and 2013 [29] to determine the risk of CHIKV transmission. As DENV is transmitted by the same vectors as CHIKV, and is subject to similar dynamics, this is likely to represent a rational, although preliminary, estimate of establishment risk [2]. As an indication of the likely intensity of CHIKV transmission if the virus does become established, we also estimated an average DENV incidence rate for each municipality from the same dataset.

## Results

### Characterization of CHIKV cases in Brazil

Table 1 summarizes the demographic characteristics of the 68 patients with confirmed CHIKV infection whose date of symptom onset was between 8 April and 15 September, 2014 (further details in Additional file 1: Table S1). Travel history was known for 67 (99%) patients. Forty-one (60%) reported travelling or residing abroad (defined as imported cases), 21 (31%) were Brazilian military and missionary personnel that had returned from Haiti, 32% travelled to Caribbean islands, and 6 (15%) to other South American countries. The remaining 27 cases were defined as autochthonous.

**Table 1:** **Demographic characteristics of CHIKV confirmed cases in Brazil 2014**

	**Number of cases (%)**
**Age (years)**	
0–19	8 (11.8%)
20–39	39 (57.4%)
40–59	15 (22.1%)
60–70	5 (7.4%)
Unknown	1 (1.5%)
**Sex**	
Female	30 (60%)
Male	20 (40%)
Unknown	18 (27%)
**State of notification**	
Amapa	16 (23.5%)
Bahia	14 (20.6%)
Ceara	5 (7.4%)
Brasilia	2 (2.9%)
Goiania	2 (2.9%)
Maranhao	1 (1.5%)
Para	1 (1.5%)
Paraná	2 (2.9%)
Rio de Janeiro	3 (4.4%)
Rio Grande do Sul	2 (2.9%)
Roraima	5 (7.4%)
Sao Paulo	15 (22.1%)
**Country of origin**	
Brazil	65 (95.6%)
Outside Brazil	3 (4.4%)
**Transmission cases**	
Imported	41 (60.3%)
Autochthonous	27 (39.7%)
**Place of infection (Autochthonous)**	
Amapa state	13 (48.2%)
Bahia state	14 (51.8%)
**Place of infection (Imported)**	
Haiti^a^	21 (51.2%)
French Guiana	1 (2.4%)
British Guiana	2 (4.9%)
Dominican Republic^b^	10 (24.4%)
Guadalupe^c^	2 (4.9%)
Venezuela	3 (7.3%)
Unknown^d^	2 (4.9%)

Data was collated between April and September 2014 from the Evandro Chagas Institute and reports from the National Program for Dengue Control. Patient’s epidemiological and clinical details can be found in Additional file 1: Table S1.

^a^Includes two travellers from Haiti; ^b^Includes one case that also travelled to Haiti; ^c^Includes one traveller from Guadalupe; ^d^Includes one patient that was infected in an unspecified Caribbean island.

**Figure 1:**
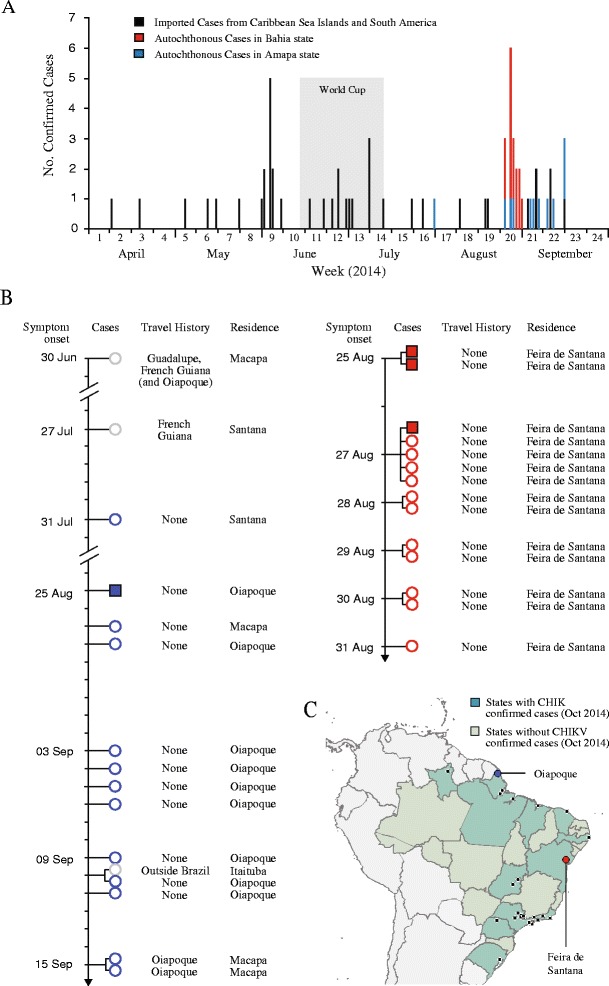
Timeline of laboratory confirmed CHIKV cases in Brazil 2014. Shown are imported (black) and autochthonous (red and blue) CHIKV cases in Brazil between April and September 2014 **(A)**. As of September, autochthonous cases had been reported in Feira de Santana, a municipality in Bahia state, north-eastern Brazil (red) and in Amapa state, north Brazil, bordering French Guiana. Also shown is the timeline of confirmed CHIKV in Amapa and Bahia state **(B)**, along with patients’ travel history and municipality of residence. Grey circles indicate imported cases, while blue and red circles indicate localized transmission. Filled squares indicate patient samples whose viruses were sequenced. **(C)** Map of confirmed cases and federal states with confirmed cases.

The number, timeline, and geographic location of cases are shown in Figure 1. Local transmission was restricted to Amapa state (48%; with 70% of these being reported in the Oiapoque municipality) and Bahia state (52%). There was a rise in the number of confirmed autochthonous cases during the last week of August 2014, but no significant difference (unpaired *t*-test two sided *P* value = 0.72) in imported cases during the month of the World Cup event.

### Asian and ECSA genotypes in Brazil

**Figure 2:**
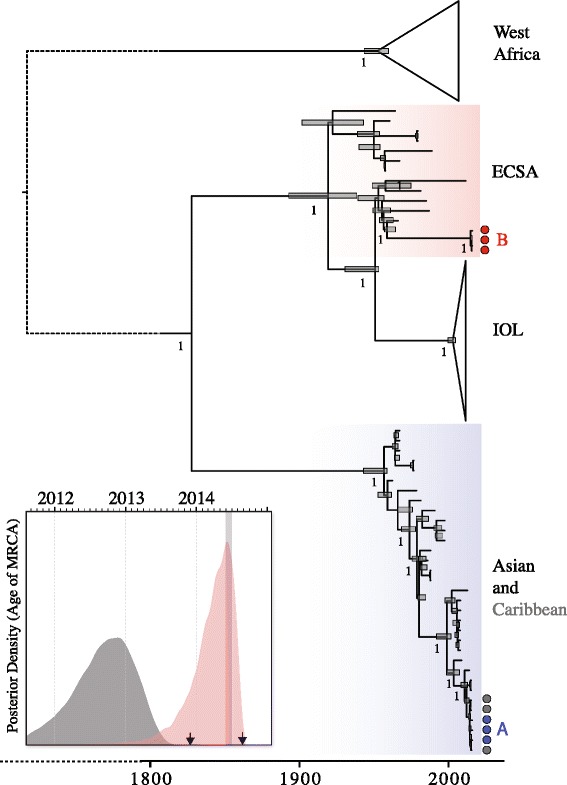
Dated phylogenetic reconstruction of CHIKV including Brazilian genomes. The genotypes involved in autochthonous Brazilian transmission in Oiapoque and Feira de Santana are highlighted in blue **(A)** and red **(B)**, respectively. On the right, isolates sampled in Feira de Santana and Oiapoque are depicted as red and blue circles, whilst isolates from the Caribbean outbreak are shown in grey circles. The inset shows the posterior density of the age of the most recent common ancestor (MRCA) of the Caribbean outbreak (**A**, grey) and the Feira de Santana cluster (**B**, red). The arrows highlight the dates of the first notifications of CHIKV cases in the Caribbean and in Feira de Santana, respectively. The index case of the ECSA genotype in Brazil arrived in Feira de Santana in June (grey bar). A ML tree with tip information can be found in Additional file 1: Figure S1.

The time-scaled phylogeny in Figure 2 includes the six Brazilian CHIKV genomes reported here (2 imported, 4 autochthonous). Both imported cases (P25, P34) plus the Oiapoque autochthonous case (P37) belonged to the Asian genotype of CHIKV. Patient P25 resides in Recife and travelled in June to the Dominican Republic. Patient P34 travelled from Guadeloupe to Belem, Para state, in August. Patient P37 resides in Oiapoque, Amapa state, did not travel abroad, and exhibited symptoms at the end of August 2014. The three Asian genotype isolates clustered closely with available genomes from the Caribbean outbreak (from Saint Martin and British Virgin Islands). The CHIKV genome from patient P37 differed from the Saint Martin isolate by 7 mutations.

We discovered and confirmed a separate CHIKV introduction to Brazil in Feira de Santana, which comprises ECSA genotype infections, a CHIKV genotype previously undetected in the Americas (Figure 2). ECSA genotype genomes were obtained from three patients (P36, P38, P39) that had not travelled abroad. Specifically, the three genome sequences were near-identical, form a phylogenetic cluster with maximum support (Figure 2), and were proximate in time and space. This supports local transmission derived from a single viral importation. The Brazilian ECSA lineage is most closely related to a CHIKV genome isolated in 1962 in Angola (Figure 2, Additional file 1: Figure S1). Similar conclusions were obtained with a larger E1 envelope dataset (Additional file 1: Figure S2). The ECSA lineage index case arrived in Feira de Santana from Angola in June 2014 and three contacts of the index case reported CHIKV-like symptoms. None of the six genomes sequenced here contain the A226V (E1 protein) or the L210Q (E2 protein) mutations that increase CHIKV transmissibility and persistence in *Ae. albopictus* [30]. The genomic evolutionary rate of the Asian genotype was estimated at 4.71 (95% BCI: 3.84–5.65) nucleotide substitutions per year, higher than that of the ECSA genotype (2.31, 95% BCI: 1.89–2.71), as previously noted [31]. We estimate that the common ancestor of CHIKV in the Caribbean existed around 23 August, 2012 (95% BCI: 3 October 2011 to 2 June 2013). We cannot estimate the date of introduction of the Asian genotype to Oiapoque because only one non-imported genome (P37) was available. However, we can date the common ancestor of the Brazilian ESCA genomes, estimated to be around March 6, 2014 (95% BCI: 29 July 2013, 15 August 2014) (Figure 2). Notably, these uncertainty intervals include the arrival date (June 2014) of the index case to Feira de Santana.

### Estimating risks of importation and establishment in Brazil

**Figure 3:**
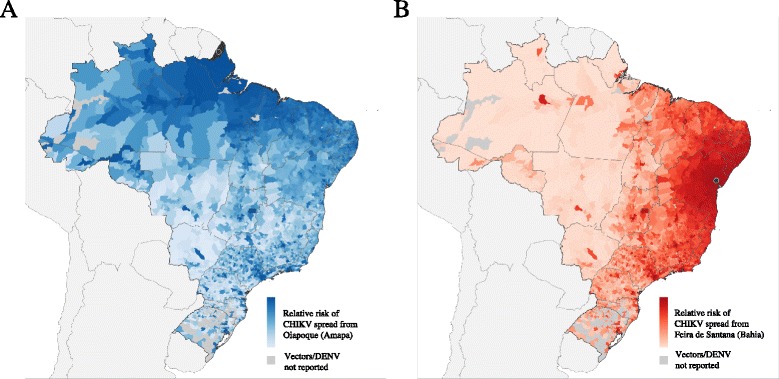
Risk maps for CHIKV spread from Oiapoque and Feira de Santana, Brazil. Shown is the relative risk of CHIKV spread from Oiapoque **(A)** and Feira de Santana **(B)**. Black circles denote the locations of these municipalities.

Modelling of human mobility predicted different geographic patterns of CHIKV importation risk from Oiapoque and Feira de Santana (Figure 3). A total of 5,172 of the 5,494 municipalities in Brazil have reported the presence of either *Ae. aegypti* or *Ae. albopictus*, or DENV transmission. These municipalities are home to the vast majority (98.6%) of Brazil’s population. Whilst only a subset of people in these regions are likely to be at risk of CHIKV infection, the public health impact of the virus is likely to be significant should it become established. We estimate that 2,093 (38.1%) municipalities in Brazil are in the top 50^th^ percentile of relative risk of importation from both Oiapoque and Feira de Santana.

**Figure 4:**
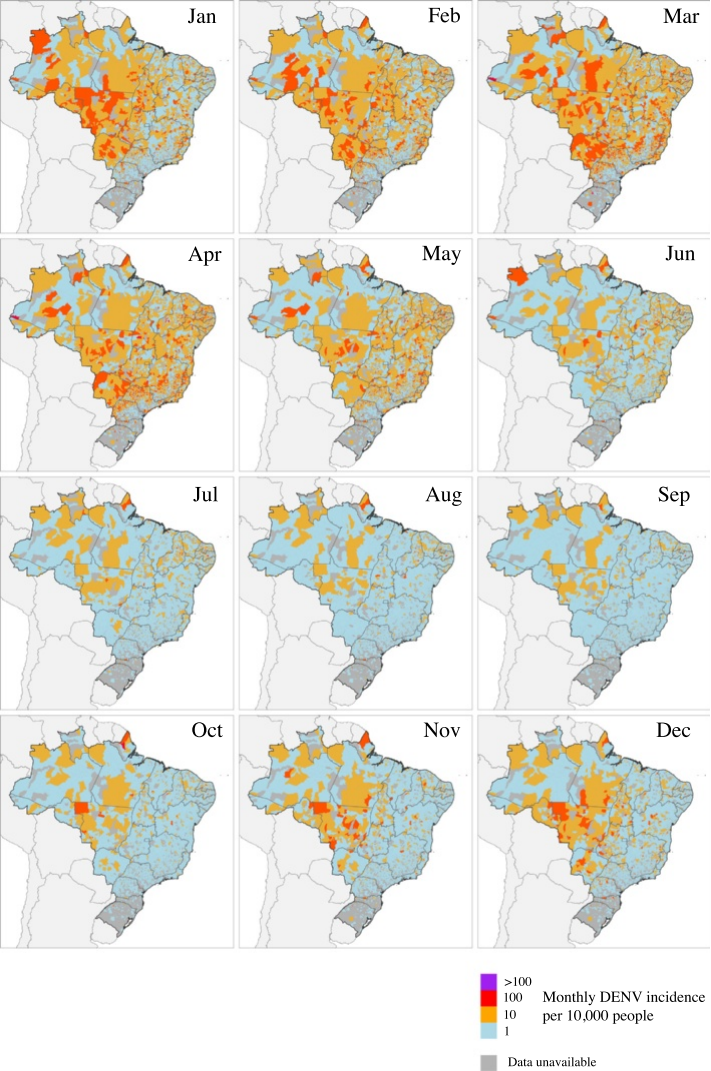
Retrospective analysis of dengue virus transmission to identify municipalities where CHIKV is expected to circulate. Spatial dengue virus monthly incidence (averaged between 2001 and 2013) is shown in Brazilian municipalities.

Of the 35 municipalities in the top 95^th^ percentile of importation risk from both Oiapoque and Feira de Santana, 57% are located in the Northeast and 31% in the Southeast region, which includes Rio de Janeiro and Sao Paulo (Additional file 1: Table S2). This suggests a chance of two CHIKV genotypes becoming established in the same geographical areas. Retrospective analysis of DENV transmission indicates that CHIKV transmission is expected to occur in year-round transmission cycles, with transmission peaks starting in November/December and peaking between January to April, maintained mostly in less populated areas located in the north and central areas of Brazil (Figure 4).

## Discussion

This study reports the emergence of both Asian and ECSA CHIKV genotypes in Brazil. This is the first time that an outbreak of the ECSA genotype has been reported in the Americas. Genetic and epidemiological data suggest that the ECSA genotype was introduced from Angola to Feira de Santana in June 2014, where epidemiological investigation is ongoing. CHIKV appears to be endemic in Angola [32], where thousands of Brazilians, mostly from Northeast and Southeast areas [33], work in the petroleum and mining industries. Genetic data suggests multiple introductions of the Asian genotype to Brazil from ongoing epidemics in the Caribbean and South America. Surveillance data suggests autochthonous transmission in Oiapoque began by early September 2014 [8], and we posit that the strain circulating there was imported from French Guiana, a country bordering Oiapoque that has reported a steady increase in autochthonous cases since January 2014 [8].

The clinical picture of patients with confirmed CHIKV infection included fever, arthralgia, rash, myalgia, and headache, symptoms similar to DENV infection, which is endemic in Brazil. The fact that we observe two different strains is sufficient to demonstrate that the epidemics in Oiapoque and Feira de Santana resulted from separate introductions and this result is robust to the number of sequences obtained. Our genetic estimate of the date of introduction of the ECSA genotype to Feira de Santana has an upper bound of 15 August, 2014, suggesting that the CHIKV surveillance network in Brazil was able to detect early cases of this introduction. Moreover, the confirmation of ~7 (3 to 13) importations of CHIKV to Brazil per month implies that Brazil is at risk of additional importations from endemic regions and that CHIKV may be exported from Brazil to other locations.

It is possible that the sustained transmission described here in results from the introduction of CHIKV strains into a location with appropriate vector abundance and during a period with appropriate climate conditions [34], particularly during monsoon rainfall period and high temperatures [35]. Some additional observations deserve further investigation. Prediction of future transmission of CHIKV within Brazil indicates that the geographic ranges of the current Asian and ECSA lineages will likely overlap and that nearly 99% of the population in Brazil may be at risk for CHIKV infection. There is no population immunity to CHIKV in Brazil, so incidence is expected to increase [36]. Training health care workers for differential diagnosis of DENV and CHIKV, increased availability of diagnostic tests for patient sera and mosquitoes, as well as educational programs and a national active surveillance system are needed to control CHIKV spread [3]. A small percentage of those living in rural northern and western areas of the country have antibodies to Mayaro virus (MAYV), a related alphavirus associated with Haemagogus mosquitoes [34]. Cross-reactivity between the antigenically-related MAYV and CHIKV [37] has been reported [38] but it is unknown if prior exposure to MAYV provides protection against CHIKV. As CHIKV genotypes are more similar to each other than to MAYV it seems probable that immunity against one CHIKV genotype will provide at least partial protection against another [39].

We follow international guidance and evaluate CHIKV importation risk using data on past DENV transmission [2]. The similarity of DENV and CHIKV transmission cycles [9] suggests that CHIKV may become endemic in several Brazilian municipalities, with transmission peaks occurring between January and April (Figure 4). Risk of CHIKV transmission is driven by a range of factors, including the presence and local abundance of suitable vector species, environmental variables such as ambient temperature, and socioeconomic factors. Whilst few of these factors are well understood or quantified for Brazil, many are likely to be shared with DENV, and it is known that the CHIKV-competent *Ae. aegypti* and *Ae. albopictus* vectors [40] are widespread in Brazil. More specifically, *Ae. aegypti* is more dispersed in Brazil with higher incidence in northern, north-eastern, central-eastern regions and less frequent in southern Brazil, due to a cooler climate [41]. In contrast, *Ae. albopictus* has high incidence in subtropical areas, more specifically in southern areas of the country [28]. Although our index of introduction risk (Figure 3) indicates the municipalities in Brazil at greatest risk for CHIKV transmission, and can be used to prioritise disease surveillance, we cannot estimate the absolute number of importations expected in each location over a given time frame because the relevant data on human mobility within Brazil are currently unavailable. This could be obtained from, for example, anonymised cell phone call records [42] or through collating existing microcensus data.

The ECSA outbreak in Feira de Santana persists and is disseminating to other regions in Brazil – and potentially to locations outside the country. Therefore, continued genetic surveillance will be necessary to investigate whether the ECSA genotype will acquire the A226V mutation in the viral E1 protein that can increase viral transmission in *Ae. albopictus* and which was associated with explosive CHIKV outbreaks in the Indian Ocean nations and Indian subcontinent [4]. Other scenarios are also possible: the Asian CHIKV genotype may become dominant in tropical regions of Brazil, where *Ae. aegypti* is well established, and the ECSA genotype in subtropical and more temperate regions, where *Ae. albopictus* is more abundant. The ECSA genotype has acquired mutations that increase transmissibility and persistence in *Ae. albopictus* on multiple independent occasions [3], whereas the Asian genotype appears less able to accrue such adaptations. In the long term, both genotypes could potentially disappear from the region if levels of human population immunity increase sufficiently. Alternatively, CHIKV could establish an enzootic cycle in the region with sporadic human epidemics. This pattern has been seen in Africa [31] and potentially in Southeast Asia [43], where there is some evidence of a sylvatic CHIKV transmission cycle involving non-human primates and forest-dwelling mosquitoes, similar to that observed for sylvatic yellow fever virus, which was introduced into tropical Americas from Africa with the slave trade and is still endemic in the Amazon and Orinoco River basins [44]. DENV serotypes also exhibit sylvatic cycles in some areas of Southeast Asia and Africa, but this has not been yet observed in the Americas.

## Conclusions

In summary, this is the first report of CHIKV emergence in Brazil. Remarkably, we detected for the first time, ongoing transmission of the ECSA CHIKV genotype in the Americas. With CHIKV transmission established in highly connected regions in the north and northeast of Brazil and with the peak suitability season fast approaching, now is the time for preventative action. Effective, targeted, and sustained viral and case surveillance and vector control measures have the potential to avert invasion of CHIKV and avoid the overwhelming of one of the world’s largest healthcare systems.

## Additional file

Additional file 1:
**Methodological details for sequencing, genetic and spatial data analysis, and supplementary tables and figures.**

## Abbreviations

BCI: Bayesian credible intervals
CHIKV: Chikungunya virus
DENV: Dengue virus
ECSA: East-Central-South-African
IOL: Indian Ocean lineage
MAYV: Mayaro virus
MCMC: Markov Chain Monte Carlo
