# Supplementary material for: Emergence and potential for spread of Chikungunya virus in Brazil
Source: BMC Med. 2015 Apr 30;13:102. doi: 10.1186/s12916-015-0348-x (PMC4433093; doi:10.1186/s12916-015-0348-x)
Supplement: Additional file 1: — Methodological details for sequencing, genetic and spatial data analysis, and supplementary tables and figures. [file 12916_2015_348_MOESM1_ESM.pdf]

Additional Files

Emergence and Potential for Spread of Chikungunya Virus in Brazil

Marcio Roberto Teixeira Nunes[1]*, Nuno Rodrigues Faria[2]*, Janaina Mota de Vasconcelos[1]*, Nick Golding, Moritz U. G. Kraemer, Layanna Freitas de Oliveira, Raimunda do Socorro da Silva Azevedo, Daisy Elaine Andrade da Silva, Eliana Vieira Pinto da Silva, Sandro Patroca da Silva, Valéria Lima de Carvalho, Giovanini Evelin Coelho, Ana Cecília Ribeiro Cruz, Sueli Guerreiro Rodrigues, Joao Lídio da Silva Gonçalves Vianez Jr, Bruno Tardelli Diniz Nunes, Jedson Ferreira Cardoso, Robert B. Tesh, Simon I. Hay, Oliver G. Pybus*, and Pedro Fernando da Costa Vasconcelos*[4].

‡**Correspondence to**:

Marcio Roberto Teixeira Nunes, Center for Technological Innovation,

Evandro Chagas Institute, Ministry of Health, Brazil.

Contents

Page

1. Methodological details

1.1. Viral Assembly Procedure 3

1.2. Accession numbers for the global CHIKV full genome dataset 3

1.3. Accession numbers for the global CHIKV envelope genome dataset 3

1.4. Predicting CHIKV importation and establishment risk 6

2. Supplementary Tables

2.1 Table S1 7

2.2 Table S2 8

3. Supplementary Figures

3.1 Figure S1. Full genome ML phylogeny of CHIKV 9

3.2 Figure S2. Envelope ML phylogeny of CHIKV 10

4. References 11

1. 1. Viral Assembly Procedure

Raw reads generated by the PGM device were used for a *de novo* assembly with Mira v4.0[1]. Relevant parameters used in the assembly were as follows: minimum read length of 100 bases (mrl=100), minimum overlap of 19 bases (mo=19), use of at least 5 reads per contig (mrpc=5) and 5 passes for the whole assembly (nop=5). The closed genomes were annotated using open reading frame predictions from Geneious v 7.0[2] and BLASTX searches[3]. Finished genomes were inspected to check if length, gene number, orientation and non-coding 5' and 3' regions corresponded to what was expected for genomes of the same family.

1.2. Accession numbers for the global CHIKV full genome dataset

Accession numbers for the representative global dataset are as follows. ECSA genotype: HM045792, HM045795, HM045805, AF490259, HM045821, HM045806, HM045809, HM045822, JQ067624, HM045812, HM045784 and HM045823. Asian genotype: HM045803, EF027140, HM045813, HM045788, EF027141, HM045810, HM045814, HM045808, HM045789, HM045802, HM045796, HM045787, HM045797, HM045791, HM045800, HM045790, HE806461, FN295483, FN295484, EU703762, EU703759, EU703760, EU703761, FJ807897, KF318729, KJ451623, KJ451622, CNR20236, CNR20235, KJ451624. Indian Ocean Genotype (Because the Brazilian isolates consistently clustered outside the IOL genotype based on preliminary maximum-likelihood[4] and Bayesian phylogenetic analysis[5], we only kept a sample of the 102 IOL genotype full-length genomes): JF274082, EU372006, GU908223, FR717336, GQ428213, KJ796852, KJ796851, KJ796845, KC862329, FN295485, FN295487, GU189061, HQ456255, HQ456254, HQ456253, HQ456252, HQ456251. West African genotype: HM045816, HM045815, HM045798, HM045785, HM045786, HM045807, AY726732, HM045817, HM045820, HM045819, HM045818.

1.3. Accession numbers for the global CHIKV envelope genome dataset

Accession numbers for the global envelope dataset are as follows. KP164567, KP164568, KP164569, KP164570, KP164571, KP164572, AB857841, HM045823, AB857833, AB857825, AB857817, AB857809, AB857801, AB857793, KC524772,

KC524771, KC524770, JN711138, JN711137, JN711136, JQ886094, JQ886093, JQ886092, JQ231211, JQ231210, JN711135, JN711134, JN711133, JN711132, JN711131, JN711130, JN711129, JN711128, JN711127, JN712437, JN712436, FJ998173, FJ513678, FJ513677, FJ513676, FJ513674, FJ513672, FJ513671, FJ513670, FJ513669, FJ513668, FJ513667, FJ513666, FJ513665, FJ513664, FJ513663, FJ513662, FJ513661, FJ513660, FJ513659, FJ513658, FJ513656, FJ513655, FJ513653, FJ513652, FJ513651, FJ513650, FJ513649, FJ513648, FJ513647, FJ513646, FJ513644, FJ513643, FJ513642, FJ513641, FJ513640, FJ513639, FJ513638, FJ513636, FJ513634, FJ513633, FJ513631, FJ513630, FJ445509, FJ445508, FJ445507, FJ445506, FJ445505, FJ445504, FJ445503, FJ445501, FJ445500, FJ445499, FJ445498, FJ445497, FJ445496, FJ445495, FJ445494, FJ445493, FJ445492, FJ445491, FJ445490, FJ445489, FJ445488, FJ445482, FJ445481, FJ445480, FJ445479, FJ445478, FJ445477, FJ445476, FJ445475, FJ445474, FJ445473, FJ445472, FJ445471, FJ445470, FJ445469, FJ445468, FJ445467, FJ445466, FJ445465, FJ445464, FJ445462, FJ445461, FJ445460, FJ445459, FJ445458, FJ445457, FJ445456, FJ445455, FJ445454, FJ445453, FJ445452, FJ445451, FJ445450, FJ445449, FJ445448, FJ445447, FJ445446, FJ445444, FJ445442, FJ445441, FJ445440, FJ445439, FJ445438, FJ445437, FJ445436, FJ445435, FJ445434, FJ445429, FJ445425, FJ445424, EF613344, EF613343, EF613342, FJ445486, JN661156, JN661155, JN661154, JN661153, JN661152, JN661151, JN661150, JN661149, JN661148, JF264892, JF264891, JF264890, GQ143750, GQ143749, GQ143748, GQ143747, GQ143746, GQ143745, GQ143743, GQ143742, FN295489, FJ617290, FJ617289, FJ617288, FJ617287, FJ617286, FJ617285, FJ617284, FJ617283, FJ617282, EU288003, EU288002, EU288001, EU287999, EU287998, EU287997, EU287995, EU287994, EU170527, EU170526, EU170525, EU170524, EU170523, EU244646, FN295495, FN295494, FN295493, FN295492, FN295491, FN295490, FN295488, FN295486, DQ489787, GQ996377, GQ996376, GQ996375, GQ996374, GQ996373, GQ996372, GQ996371, GQ996370, JX839809, JX839808, JX839807, JX839806, JX839805, JX839804, JX839803, JX839802, JX839824, JX839823, JX839822, JX839821, JX839820, JX839819, JX839818, GQ143744, JX839784, JX839783, JX839782, JX839781, JX839780, JX839779, JX839778, JX839777, JQ013707, JQ013706, JQ013705, JQ013704, JQ013703, JQ013702, JQ013701, JQ013700, JQ013699, JQ013693, JQ013692, JQ013691, JQ013690, JQ013689, JQ013687, AB678695, AB678694, AB678693, AB678692, AB678691, AB678690, AB678689, AB678688, AB678687, AB678686, AB678685, AB678684, AB678683, AB678682, AB678681, AB678680, AB678679, AB678678, AB678677, GQ229489, GQ229488, GQ229487, GQ229486, GU969242, FJ445487, FJ445485, FJ445483, EU441883, EU441882, KC731586, KC731585, KC731584, KC731583, KC731582, KC731581, JQ943720, JQ943719,

JQ943718, JQ943717, JQ943716, JQ943715, JQ943714, JQ943713, JQ943712, JQ943711, JQ943710, JQ943709, JQ943708, JQ943707, JQ943706, JQ943705, JQ943704, JQ943703, JQ943702, JQ943701, JQ943700, JQ943699, JQ943698, JQ943696, JQ943695, JQ943694, JQ943693, JQ943692, GQ143741, JQ943691, JQ943690, JQ943689, JQ943688, HQ529778, JQ943687, HE578162, JQ943697, KC879578, KC879577, KC879576, KC879575, KC879574, KC879573, KC879572, KC879571, KC879570, KC879569, KC879568, KC879567, KC879566, KC879565, KC879564, KC879563, KC879562, KC879561, KC879560, KC879559, KC969208, KC969207, JX839829, JX839828, JX839827, JX839826, JX839825, JX839817, JX839816, JX839815, JX839814, JX839813, JX839812, JX839811, JX839810, JX839801, JX839800, JX839799, JX839798, JX839797, JX839796, JX839795, JX839794, JX839793, JX839792, JX839791, JX839790, JX839789, JX839788, JX839787, JX839786, JX839785, JX839776, JX839775, JX839774, JX839773, JX839772, JX839771, JX839770, JX839769, JX839768, JX839767, JX839766, GQ996379, GQ996378, EF555200, EF555199, EF555198, EF555197, EF555196, FJ432665, AF339485, FJ705371, FJ705370, FJ705369, CNR20236, CNR20235, HM045820, HM045819, HM045818, HM045817, HM045816, HM045807, HM045804, HM045798, HM045815, HM045785, HM045786, AY726732, HM045784, JF274082, EU037962, KF318729, JN558836, JN558835, JN558834, JX088705, DRDE-07, HM045822, HM045821, HM045814, HM045813, HM045812, HM045810, HM045809, HM045808, HM045806, HM045805, HM045803, HM045802, HM045801, HM045800, HM045799, HM045797, HM045796, SAH2123, HM045794, HM045793, HM045792, HM045791, HM045790, HM045789, HM045788, HM045787, AF490259, GU301781, GU301780, GU301779, GU908223, GQ905863, FJ445511, FJ445510, FJ445502, FJ445484, FJ445463, FJ445445, FJ445443, FJ445433, FJ445432, FJ445431, FJ445430, FJ445428, FJ445427, FJ445426, RGCB356, RGCB355, RGCB120, GQ428212, GQ428211, GQ428210, FJ000069, FJ000068, FJ000067, FJ000066, FJ000065, FJ000064, FJ000063, FJ000062, EF210157, KJ796852, KJ796851, KJ796850, KJ796849, KJ796848, KJ796847, KJ796846, KJ796845, KJ796844, KJ451624, KJ451623, KJ451622, KC862329, FN295483, FN295485, FN295487, FN295484, JQ067624, GU189061, HQ456255, HQ456254, HQ456253, HQ456252, HQ456251, GU199353, GU199352, GU199351, GU199350, FJ513679, FJ513675, FJ513673, FJ513657, FJ513654, FJ513645, FJ513637, FJ513635, FJ513632, FJ513629, FJ513628, FJ807899, FJ807898, FJ807897, FJ807896, FJ959103, EU244823, EU564335, EU564334, EF012359, EF027141, EF027140, EF027138, EF027137, EF027136, EF027135, EF027134, DQ443544, HE806461, FR717337, FR717336, EU703762, EU703761, EU703760, EU703759, AB455494, AB455493, HM159390, HM159389, HM159388, HM159387, HM159386, HM159385, HM159384.

1.4. Predicting importation and establishment risk

Under this model M_{ij}, the number of people moving from region i to j is given by:

$$M_{ij} = p\frac{\left(\frac{1-\lambda^{(n_i+s_{ij}+1)}}{n_i+s_{ij}+1} - \frac{1-\lambda^{(n_i+n_j+s_{ij}+1)}}{n_i+n_j+s_{ij}+1}\right)}{\frac{1-\lambda^{(n_i+1)}}{n_i+1}},$$

where n_i and n_j are the populations at regions i and j respectively, s_{ij} is the total population living in all regions no further from region i than region j is, and p and λ are free parameters bounded on the unit interval which respectively govern the fraction of the population moving and the shape of the resulting distribution.

2.1 Supplementary Table S1.

Epidemiological and clinical characteristics of CHIKV confirmed cases.

Patient code (Acc. No)	Age, y (1)	Municipality of residence, 2 (State, 3)	Simplified	Country of origin	Diagnostic exam	Date of symptoms onset (SO)	Date of sample collection (SC)	SO-SC (d)	DENV serology	Location of travel	Date of travel (4)
P1	50-59	Porto Alegre (Rio Grande do Sul) (D)	Imported	Haiti	ELISA	08-Apr	24-Jun	77	-	Brazil (Fortaleza and Rio Grande do Sul)	early April
P2	60-69	Porto Alegre (Rio Grande do Sul) (D)	Imported	Haiti	ELISA	18-Apr	24-Jun	67	-	Brazil (Fortaleza and Rio Grande do Sul)	early April
P3	20-29	Nova Friburgo (Rio de Janeiro)	Imported	Brazil	ELISA	04-May	30-May	26	Positive	Haiti	early May
P4	20-29	Monte Azul Paulista (Sao Paulo)	Imported	Brazil	ELISA	12-May	22-May	-	-	Haiti	-
P5	30-39	Arraial do Cabo (Rio de Janeiro)	Imported	Brazil	ELISA	15-May	03-Jun	19	-	Haiti	
P6	40-49	Porto Alegre (Sao Paulo)	Imported	Brazil	ELISA/RT-qPCR	23-May	06-Jun	14	-	Haiti	-
P7	30-39	Maringá (Paraná) (C)	Imported	Brazil	ELISA	31-May	18-Jun	18	Negative	Haiti	-
P8	20-29	Itu (Sao Paulo)	Imported	Brazil	ELISA/RT-qPCR	01-Jun	05-Jun	4	Negative	Haiti	-
P9	30-39	Maringá (Paraná) (C)	Imported	Brazil	ELISA	01-Jun	18-Jun	17	Negative	Haiti	-
P10	20-29	Campinas (Sao Paulo)	Imported	Brazil	ELISA/RT-qPCR	03-Jun	05-Jun	2	Negative	Haiti	-
P11	20-29	Sao Vicente (Sao Paulo)	Imported	Brazil	ELISA/RT-qPCR	03-Jun	05-Jun	2	Negative	Haiti	-
P12	40-49	Taubate (Sao Paulo)	Imported	Brazil	ELISA/RT-qPCR	03-Jun	05-Jun	2	Negative	Haiti	-
P13	20-29	Hortolandia (Sao Paulo)	Imported	Brazil	ELISA/RT-qPCR	03-Jun	05-Jun	2	Negative	Haiti	-
P14	30-39	Manaus (Sao Paulo)	Imported	Brazil	ELISA/RT-qPCR	03-Jun	05-Jun	2	Negative	Haiti	-
P15	20-29	Campinas (Sao Paulo)	Imported	Brazil	ELISA/RT-qPCR	04-Jun	05-Jun	1	Negative	Haiti	-
P16	20-29	Sao Vicente (Sao Paulo)	Imported	Brazil	ELISA/RT-qPCR	04-Jun	06-Jun	2	-	Haiti	-
P17	20-29	Campinas (Sao Paulo	Imported	Brazil	ELISA/RT-qPCR	07-Jun	07-Jun	0	-	Haiti	-
P18	20-29	São Paulo (Sao Paulo)	Imported	Brazil	ELISA/RT-qPCR	17-Jun	15-Jul	28	-	Dominican Rep.	-
P19	30-39	Rio de Janeiro (Rio de Janeiro)	Imported	Brazil	ELISA/RT-qPCR	22-Jun	24-Jun	2	-	Dominican Rep.	-
P20	20-29	São Luiz (Maranhao)	Imported	Brazil	ELISA	25-Jun	-	-	-	Haiti	-
P21	50-59	Brasilia (Distrito Federal)	Imported	Brazil	ELISA	27-Jun	02-Jul	-	-	Haiti	early June
P22	40-49	Goiania (Goias)	Imported	Brazil	ELISA	27-Jun	24-Jul	27	-	Dominican Rep.	late June
P23	10-19	Macapa (Amapa)	Imported	Brazil	ELISA	30-Jun	02-Jul	2	Negative	Guadalupe, French Guiana and Brazil (Oiapoque)	late June
P24	40-49	Brasilia (Distrito Federal)	Imported	Brazil	ELISA	01-Jul	04-Jul	3	-	Dominican Rep.	late June
**P25 (KP164571)**	**20-29**	**Recife (Pernambuco)**	Imported	**Brazil**	**IV-C6/36**	**02-Jul**	**03-Jul**	**1**	**-**	**Dominican Rep.**	**early June**
P26	30-39	Fortaleza (Ceara)	Imported	Brazil	ELISA/RT-qPCR	08-Jul	19-Jul	11	-	Dominican Rep.	-
P27	20-29	Fortaleza (Ceara)	Imported	Brazil	ELISA	08-Jul	21-Jul	13	-	Dominican Rep.	-
P28	50-59	Fortaleza (Ceara)	Imported	Brazil	ELISA	08-Jul	21-Jul	13	-	Dominican Rep.	-
P29	20-29	Fortaleza (Ceara)	Imported	Brazil	ELISA	13-Jul	21-Jul	8	-	Dominican Rep.	-
P30	60-69	Araçoiama (Sao Paulo)	Imported	Brazil	ELISA/RT-qPCR	23-Jul	24-Jul	1	-	Dominican Rep. and Haiti	-
P31	10-19	Santana (Amapa)	Imported	Brazil	ELISA	27-Jul	31-Jul	4	-	French Guiana	-
P32	10-19	Santana (Amapa)	Autochthonous	Brazil	ELISA/RT-qPCR	31-Jul	31-Jul	0	-	Did not travel outside Brazil	-
P33	50-59	Goiânia (Goias)	Imported	Brazil	ELISA/RT-qPCR	09-Aug	-	-	-	Caribbean region	-
**P34 (KP164572)**	**40-49**	**Belem (Para)**	Imported	**Guadalupe**	**IV-C6/36**	**18-Aug**	**21-Aug**	**3**	**-**	**Guadalupe**	**-**
P35	20-29	Boa Vista (Roraima)	Imported	Brazil	RT-qPCR	19-Aug	22-Aug	3	-	Venezuela	-
**P36 (KP164568)**	**60-69**	**Feira de Santana (Bahia)**	Autochthonous	**Brazil**	**IV-C6/36**	**25-Aug**	**26-Aug**	**1**	**-**	**None**	**-**
**P37 (KP164567)**	**30-39**	**Oiapoque (Amapa)**	Autochthonous	**Brazil**	**IV-C6/36**	**25-Aug**	**28-Aug**	**3**	**-**	**None**	**-**
**P38 (KP164570)**	**30-39**	**Feira de Santana (Bahia)**	Autochthonous	**Brazil**	**IV-C6/36**	**19-Aug**	**03-Sep**	**9**	**-**	**None**	**-**
**P39 (KP164569)**	**20-29**	**Feira de Santana (Bahia) (A)**	Autochthonous	**Brazil**	**IV-C6/36**	**27-Aug**	**28-Aug**	**1**	**-**	**None**	**-**
P40	30-39	Feira de Santana (Bahia)	Autochthonous	Brazil	RT-qPCR	27-Aug	28-Aug	1	-	None	-
P41	20-29	Feira de Santana (Bahia)	Autochthonous	Brazil	ELISA	27-Aug	03-Sep	7	-	None	-
P42	60-69	Feira de Santana (Bahia)	Autochthonous	Brazil	ELISA	27-Aug	03-Sep	7	-	None	-
P43	50-59	Feira de Santana (Bahia)	Autochthonous	Brazil	ELISA	27-Aug	04-Sep	8	-	None	-
P44	50-59	Macapa (Amapa)	Autochthonous	Brazil	ELISA	27-Aug	09-Sep	13	-	None	-
P45	20-29	Feira de Santana (Bahia) (B)	Autochthonous	Brazil	ELISA	28-Aug	04-Sep	7	-	None	-
P46	30-39	Feira de Santana (Bahia) (A)	Autochthonous	Brazil	ELISA	28-Aug	04-Sep	7	-	None	-
P47	0-9	Oiapoque (Amapa)	Autochthonous	Brazil	ELISA	28-Aug	09-Sep	11	-	None	-
P48	-	Feira de Santana (Bahia) (B)	Autochthonous	Brazil	ELISA/RT-qPCR	29-Aug	03-Sep	5	-	None	-
P49	70-79	Feira de Santana (Bahia)	Autochthonous	Brazil	ELISA	29-Aug	04-Sep	6	-	None	-
P50	30-39	Feira de Santana (Bahia)	Autochthonous	Brazil	ELISA	30-Aug	04-Sep	5	-	None	-
P51	50-59	Feira de Santana (Bahia) (A)	Autochthonous	Brazil	RT-qPCR	30-Aug	04-Sep	5	-	None	-
P52	0-9	Feira de Santana (Bahia) (A)	Autochthonous	Brazil	ELISA	31-Aug	04-Sep	4	-	None	-
P53	0-9	Boa Vista (Roraima)	Imported	Brazil	ELISA	02-Sep	09-Sep	7	-	Venezuela	early Septermber
P54	30-39	Oiapoque (Amapa)	Autochthonous	Brazil	ELISA	03-Sep	19-Sep	16	-	None	-
P55	20-29	Oiapoque (Amapa)	Autochthonous	Brazil	ELISA	04-Sep	15-Sep	11	-	None	-
P56	20-29	Oiapoque (Amapa)	Autochthonous	Brazil	ELISA	05-Sep	11-Sep	6	-	Brazil (Santa Isabel, Piquia)	-
P57	20-29	Boa Vista (Roraima)	Imported	Brazil	ELISA	05-Sep	17-Sep	12	-	Venezuela	late August
P58	30-39	Oiapoque (Amapa)	Autochthonous	Brazil	ELISA	06-Sep	15-Sep	9	-	None	-
P59	40-49	Oiapoque (Amapa)	Autochthonous	Brazil	ELISA	09-Sep	15-Sep	6	-	None	-
P60	40-49	Itaituba (Amapa)	Imported	Brazil	RT-qPCR	10-Sep	11-Sep	1	-	-	-
P61	40-49	Oiapoque (Amapa)	Autochthonous	Brazil	RT-qPCR	10-Sep	12-Sep	1	-	Brazil (Porto Grande, Cupixi, Macapa, Sao Paulo)	late August
P62	20-29	Oiapoque (Amapa)	Autochthonous	Brazil	RT-qPCR	11-Sep	12-Sep	1	-	None	-
P63	20-29	Boa Vista (Roraima)	Imported	Brazil	RT-qPCR	15-Sep	20-Sep	5	-	British Guiana	early September
P64	0-9	Macapa (Amapa)	Autochthonous	Brazil	ELISA	15-Sep	22-Sep	7	-	Brazil (Oiapoque)	early September
P65	20-29	Macapa (Amapa)	Autochthonous	Brazil	ELISA	15-Sep	22-Sep	7	-	Brazil (Oiapoque)	early September
P66	30-39	Praia Grande (Sao Paulo)	Imported	Brazil	ELISA/RT-qPCR	-	06-Jun	-	-	Haiti	-
P67	20-29	Campinas (Sao Paulo	Imported	Brazil	ELISA/RT-qPCR	-	06-Jun	-	-	Haiti	-
P68	0-9	Boa Vista (Roraima)	Imported	Brazil	ELISA	-	20-Sep	-	-	British Guiana	-

Notes:

1. Ages have been grouped into decades to preserve anonymity.
2. A, B, C and D reside in the same street or address and are presumed clusters of CHIKV infection.
3. Federal state of notification.
4. Dates of travel have been summarized to the nearest fortnight to ensure anonymity.

2.2 Supplementary Table S2.

The 35 municipalities at higher risk of CHIKV importation from both Oiapoque and Feira de Santana municipalities.

Municipality	Federal State	Country Region
Brasilia	Distrito Federal	Central-West
Goiania	Goias	Central-West
Manaus	Amazonas	North
Belem	Para	North
Maceio	Alagoas	Northeast
Feira de Santana	Bahia	Northeast
Salvador	Bahia	Northeast
Caucaia	Ceara	Northeast
Crato	Ceara	Northeast
Fortaleza	Ceara	Northeast
Sao Luis	Maranhao	Northeast
Bayeux	Paraiba	Northeast
Campina Grande	Paraiba	Northeast
Joao Pessoa	Paraiba	Northeast
Santa Rita	Paraiba	Northeast
Abreu E Lima	Pernambuco	Northeast
Camaragibe	Pernambuco	Northeast
Jaboatao Dos Guararapes	Pernambuco	Northeast
Paulista	Pernambuco	Northeast
Petrolina	Pernambuco	Northeast
Recife	Pernambuco	Northeast
Teresina	Piaui	Northeast
Natal	Rio Grande Do Norte	Northeast
Aracaju	Sergipe	Northeast
Belo Horizonte	Minas Gerais	Southeast
Contagem	Minas Gerais	Southeast
Duque de Caxias	Rio de Janeiro	Southeast
Nilopolis	Rio de Janeiro	Southeast
Rio de Janeiro	Rio de Janeiro	Southeast
Carapicuiba	Sao Paulo	Southeast
Diadema	Sao Paulo	Southeast
Guarulhos	Sao Paulo	Southeast
Maua	Sao Paulo	Southeast
Sao Caetano do Sul	Sao Paulo	Southeast
Sao Paulo	Sao Paulo	Southeast

3.1. Supplementary Figure S1.

Maximum-likelihood phylogenetic reconstruction of 76 CHIKV genomes, including the Brazilian strains. The strain names in red correspond to Brazilian isolates belonging to the ECSA genotype and the strain names in blue indicate Brazilian isolates belonging to the Asian genotype. Numbers from 0 to 1 indicate phylogenetic branch support obtained using an approximate likelihood ratio test (SH-like) in PhyML [6]. Branch lengths in units of substitutions per site.

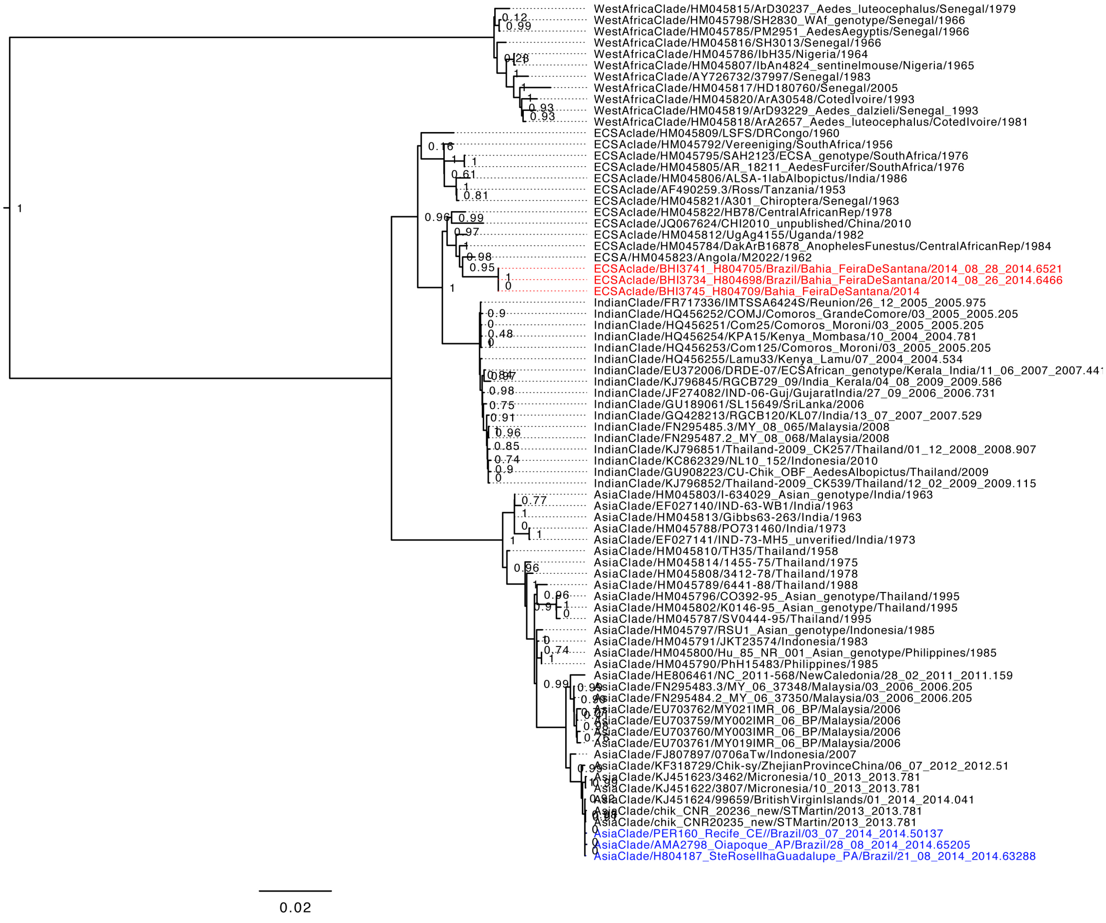

3.2. Supplementary Figure S2.

Maximum-likelihood phylogenetic reconstruction of 554 CHIKV envelope sequences, including the Brazilian strains. The strain names in red correspond to Brazilian isolates belonging to the ECSA genotype and the strain names in blue indicate Brazilian isolates belonging to the Asian genotype. Numbers from 0 to 1 indicate phylogenetic branch support obtained using an approximate likelihood ratio test (SH-like) in PhyML [6].

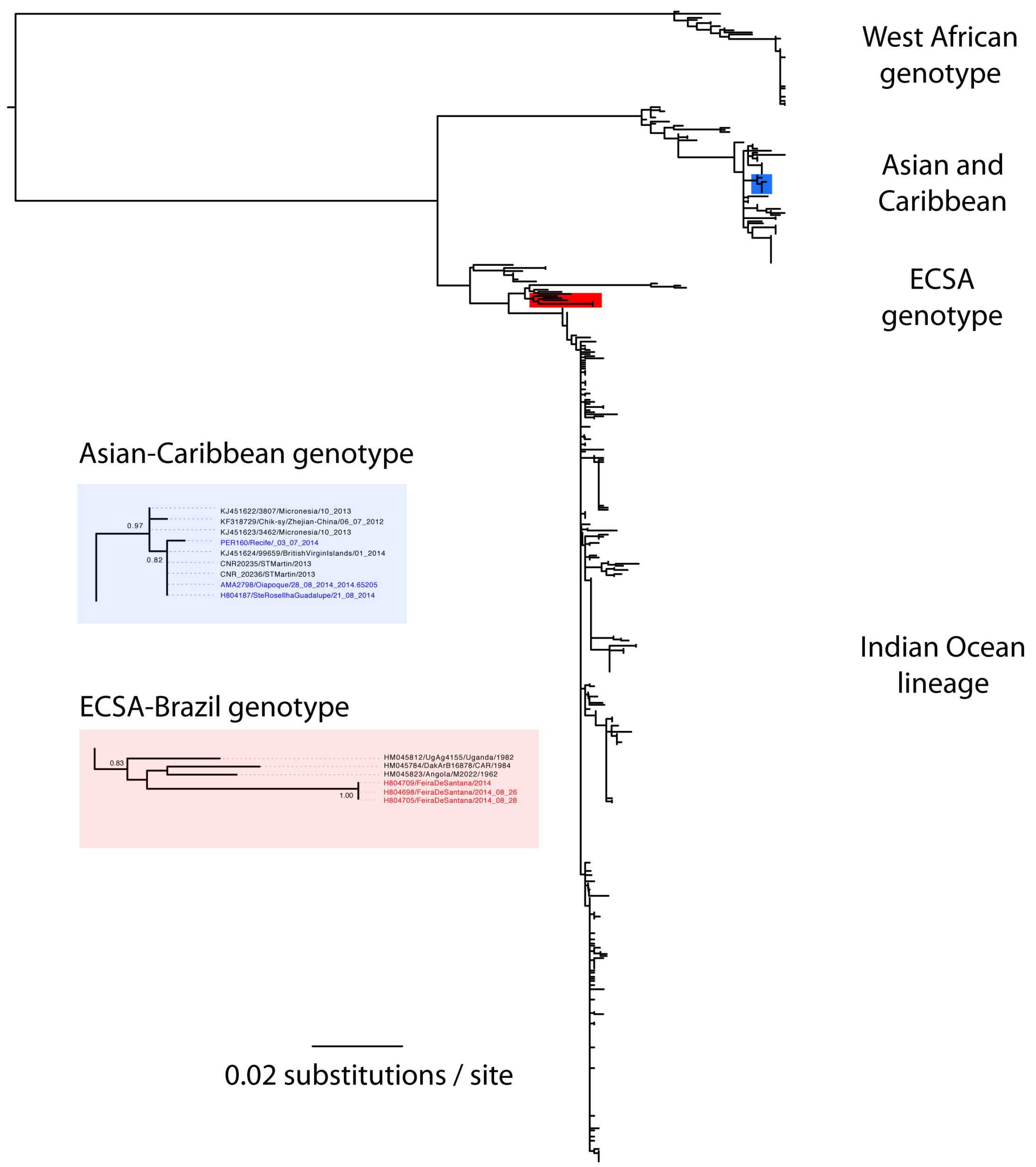

4. References

1. Chevreux B, Pfisterer, T., Drescher, B., Driesel, A. J., Muller, W. E. G., Wetter, T., Suhai, S. (2004) Using the miraEST Assembler for Reliable and Automated mRNA Transcript Assembly and SNP Detection in Sequenced ESTs. Genome Research: 1147-1159.
2. Kearse M, Moir R, Wilson A, Stones-Havas S, Cheung M, et al. (2012) Geneious Basic: an integrated and extendable desktop software platform for the organization and analysis of sequence data. Bioinformatics 28: 1647-1649.
3. Altschul SF, Gish W, Miller W, Myers EW, Lipman DJ (1990) Basic local alignment search tool. J Mol Biol 215: 403-410.
4. Guindon S, Dufayard JF, Lefort V, Anisimova M, Hordijk W, et al. (2010) New algorithms and methods to estimate maximum-likelihood phylogenies: assessing the performance of PhyML 3.0. Syst Biol 59: 307-321.
5. Drummond AJ, Suchard MA, Xie D, Rambaut A (2012) Bayesian phylogenetics with BEAUti and the BEAST 1.7. Mol Biol Evol 29: 1969-1973.
6. Gouy M, Guindon S, Gascuel O (2010) SeaView version 4: A multiplatform graphical user interface for sequence alignment and phylogenetic tree building. Mol Biol Evol 27: 221-224.
